# Comparison of Radiation Doses for Different Techniques in Fluoroscopy-Guided Lumbar Facet Medial Branch Blocks: A Retrospective Cohort Study

**DOI:** 10.3390/life14091179

**Published:** 2024-09-19

**Authors:** Mesut Bakır, Şebnem Rumeli, Mehmet Ertargın, Nurettin Teker, Mustafa Azizoğlu, Gülçin Gazioğlu Türkyılmaz

**Affiliations:** 1 Division of Pain Medicine, Department of Anesthesiology and Reanimation, Faculty of Medicine, Mersin University, Mersin 33343, Turkey; 2 Department of Anesthesiology and Reanimation, Faculty of Medicine, Mersin University, Mersin 33343, Turkey; 3 Pain Clinic, Bursa City Hospital, Bursa 16110, Turkey

**Keywords:** fluoroscopy, lumbar facet medial branch blocks, radiation exposure, radiation safety

## Abstract

Chronic lumbar facet pain is commonly treated with fluoroscopy-guided facet medial branch blocks (FMBBs). However, the associated radiation exposure of both patients and clinicians is a growing concern. This study aimed to compare radiation doses and fluoroscopy times between two techniques, i.e., oblique and posterior–anterior (PA) fluoroscopic approaches, while also examining the impact of physician experience on these metrics. A retrospective analysis was conducted on 180 patients treated at Mersin University Hospital Pain Clinic between January and July 2024. Patients were divided into two groups: 90 received the oblique technique (Group O) and 90 received the AP technique (Group A). Radiation dose and fluoroscopy time data were collected for each patient. The AP technique was associated with significantly lower radiation doses (mean 66 mGy) and shorter fluoroscopy times (mean 28 s) compared to the oblique technique (mean radiation dose of 109 mGy and fluoroscopy time of 46 s) (*p* < 0.001). Physician experience also influenced these outcomes, with more experienced physicians consistently using less radiation. The AP technique should be considered for FMBBs, as it reduces radiation exposure while maintaining procedural efficiency, highlighting the importance of experience in optimizing outcomes.

## 1. Introduction

In recent years, there has been an exponential increase in the number of imaging-guided interventional procedures, which has subsequently increased patients’ and physicians’ cumulative exposure to ionizing radiation [1]. The ALARA (“as low as reasonably achievable”) protocol was initially intended primarily for radiology departments, but it is now recognized as essential in any clinical setting where radiation is used, including anesthesiology [2]. Concerns regarding radiation exposure are well-founded, as ionizing radiation is known to carry risks such as radiation-induced tissue reactions, including erythema and radiation-induced skin injuries, as well as the potential for malignancies and genetic mutations [3]. The need for minimizing radiation exposure during these procedures is paramount, which is why strategies such as maintaining appropriate distance, minimizing exposure time, and using protective measures have been emphasized [4].

Facet joint pain is a significant contributor to chronic spinal pain and can be managed with various therapeutic techniques, including fluoroscopically guided facet medial branch blocks (FMBBs) [5,6]. Fluoroscopy-guided FMBBs can be performed using different techniques, notably the fluoroscopic oblique and anterior–posterior (AP) approaches [7]. The AP technique is generally thought to be easier to perform and is often associated with lower radiation exposure for both clinicians and patients [8].

This study aims to compare the radiation doses and fluoroscopy times associated with the fluoroscopic oblique and AP techniques in lumbar FMBBs while also examining the influence of physician experience on these metrics by comparing procedures performed by a professor and a fellow doctor. By analyzing data from a cohort of patients treated at Mersin University Hospital Pain Clinic, this study seeks to provide valuable insights into optimizing radiation safety while maintaining procedural efficacy.

## 2. Materials and Methods

### 2.1. Study Design and Setting

This retrospective cohort study was conducted at Mersin University Hospital Pain Clinic after receiving approval from the institutional ethics committee (date: 26 July 2024; number: 78017789/2793425). This study analyzed data from 180 patients who underwent fluoroscopy-guided lumbar FMBBs between January and July 2024. The primary aim of this study was to compare the radiation doses and procedure times between two fluoroscopic techniques: oblique and PA. Secondary outcomes included the impact of physician experience on these metrics.

### 2.2. Sample Size Calculation

The required sample size for this study was determined based on a power analysis conducted prior to the initiation of this study. The analysis aimed to detect a medium effect size (Cohen’s d = 0.5) with a significance level (alpha) of 0.05 and a statistical power of 90%. The decision to aim for detecting a medium effect size (Cohen’s d ≈ 0.5) was based on the anticipated clinical relevance of the differences between the oblique and PA techniques. Given the expected variability in radiation doses and fluoroscopy times, a medium effect size was chosen to detect clinically meaningful differences while maintaining a balance between sample size and study power. This calculation indicated that a minimum of 85 participants per group would be necessary to achieve adequate power. Therefore, the total sample size required for this study was 170 patients, divided equally between the two groups (Group O: oblique technique, Group A: anterior–posterior (AP) technique).

### 2.3. Patient Selection

The inclusion criteria for this study included patients aged 18–80 years who underwent lumbar FMBBs for the management of chronic facet joint pain. Patients who underwent transforaminal anterior and interlaminar epidural steroid injection in addition to lumbar facet medial branch block were also included in this study. The exclusion criteria were patients who had undergone lumbar stabilization surgery and those who had additional procedures outside the lumbar spine, such as caudal epidural steroid injection or sacroiliac joint injection. The exclusion criteria ensured the avoidance of confounding factors that might influence radiation doses or procedure times. Patients who had undergone lumbar stabilization surgery were excluded from this study because the presence of stabilization hardware could interfere with fluoroscopic imaging, potentially affecting both radiation exposure and fluoroscopy time. In contrast, patients who had undergone prior lumbar surgery without stabilization were included, as their imaging quality and procedural outcomes were not affected by such hardware.

**Figure 1:**
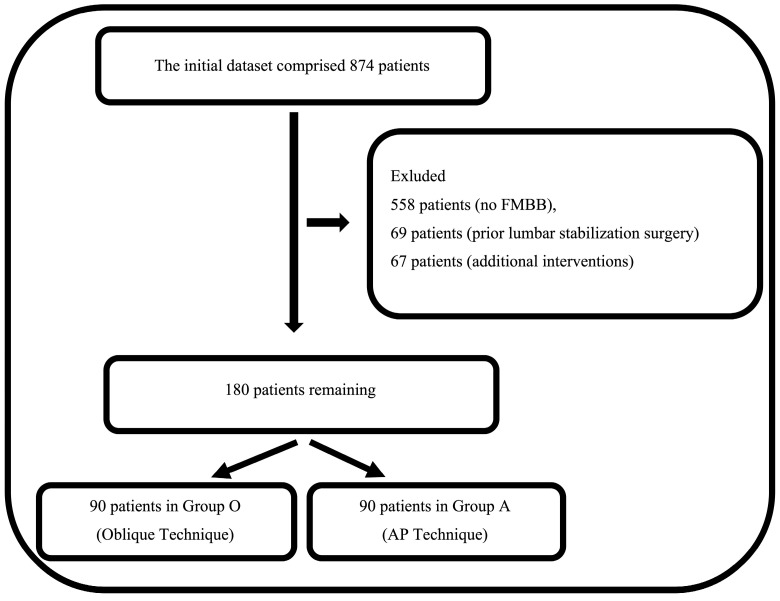
Patient flow chart.

A total of 874 patients who underwent pain interventions under fluoroscopic guidance within the specified study period were evaluated. Of these, 694 patients who did not meet the study criteria were excluded. The remaining 180 patients were then analyzed, and it was observed that 90 of these patients received lumbar FMBB using the oblique technique (Group O), while the remaining 90 patients were treated using the AP technique (Group A) (Figure 1). Patients were allocated to the oblique or posterior–anterior technique groups based on clinical considerations, including anatomical factors and the need for optimal visualization, as determined by the surgeon. While the allocation was not random, it reflected the natural decision-making process in routine clinical practice. The equal distribution of patients between the two groups (90 per group) occurred by chance and was not the result of intentional balancing. This outcome, though seemingly balanced, represents the inherent variability in clinical practice rather than a predefined strategy. This study retrospectively analyzed the techniques as they were naturally chosen in routine clinical practice.

### 2.4. Description of Techniques

#### 2.4.1. Oblique Technique

**Figure 2:**
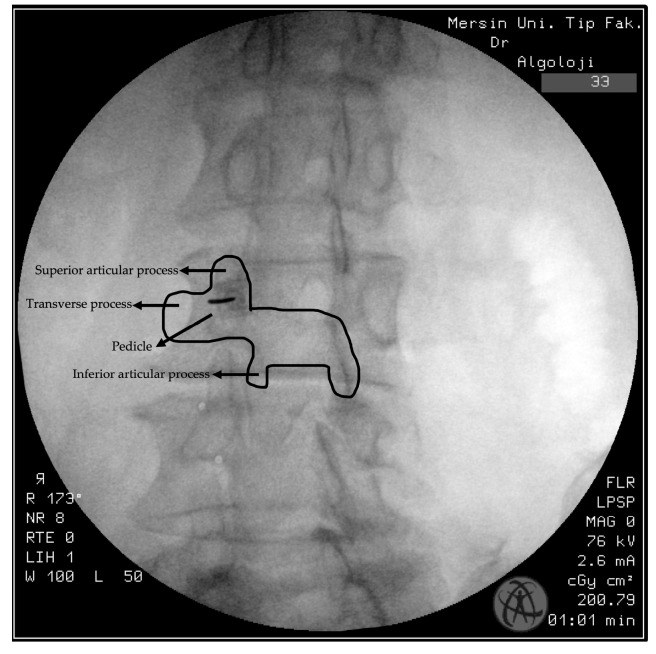
Oblique technique FMBB, “Scottie dog” view.

Patients are positioned prone in the operation room under standard fluoroscopic guidance. Once the vertebral end plates are aligned in a single line, the fluoroscope is adjusted to a 20-degree oblique position. The target, the facet medial branches, is identified by visualizing the “Scottie dog”, a well-known radiographic landmark used during FMBBs. The Scottie dog represents the target area where the facet medial branch passes, and this approach allows for precise needle placement [9]. (Figure 2).

#### 2.4.2. Anterior–Posterior (AP) Technique

**Figure 3:**
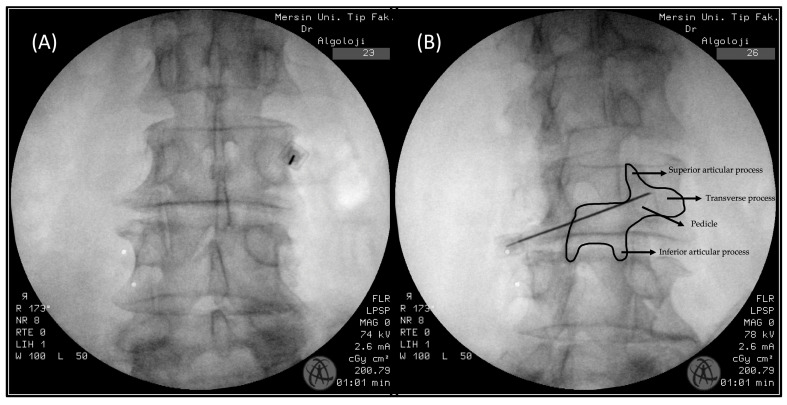
(**A**): Anterior–posterior (AP) technique FMBB fluoroscopy view. (**B**) This image was taken to ensure that the needle was in the targeted “Scottie dog” area.

In this technique, patients are also positioned prone. However, unlike the oblique technique, the fluoroscope remains in a direct AP position. The target area is the medial part of the transverse process, near the pedicle, which corresponds to the same region identified by the Scottie dog in the oblique technique [10] (Figure 3A,B).

### 2.5. Data Collection and Outcomes

The primary outcomes measured were the radiation dose and fluoroscopy time, particularly for one FMBB, as well as the total radiation dose and fluoroscopy time for the entire procedure, comparing the oblique and AP fluoroscopy techniques.

Secondary outcomes included the impact of physician experience, comparing procedures performed by a professor (M.B., S.R.) and a fellow doctor (N.R., M.E.) in terms of the aforementioned metrics. Primary and secondary outcomes were compared between the oblique and AP techniques, as well as between procedures performed by the professor and fellow doctors.

Data were collected retrospectively from patient records at our pain clinic and included patient age, sex, body mass index (BMI), the technique used, any additional interventions, the number of FMBB levels, the number of images, radiation dose, fluoroscopy time, and the physician who performed the procedure. Each procedure’s radiation dose and fluoroscopy time were recorded using the C-arm fluoroscopy unit’s built-in tracking system. Since 2014, for all patients who undergo interventions under fluoroscopic guidance, images, radiation doses, and fluoroscopy times have been systematically documented in an electronic archive. All procedures were conducted using the same fluoroscopy unit (Ziehm Solo). In our clinic, it is standard practice for the same radiology technician to operate the fluoroscopic equipment for all procedures, ensuring consistency in imaging angles and radiation settings across interventions.

If multiple procedures were performed for these patients, the fluoroscopy image count, radiation dose, and fluoroscopy time specific to the FMBB procedure were calculated by dividing the total values by the number of interventions per procedure. The standard deviations reported in this study reflect the variability in radiation doses and fluoroscopy times. Given the sufficiently large sample size (*n* = 180), the standard deviations provide a meaningful and statistically valid measure of variability.

### 2.6. Statistical Analysis

Data were analyzed using SPSS version 22. Descriptive statistics were used to summarize patient demographics and procedural details. Results were reported as mean ± standard deviation (SD), and, where applicable, 95% confidence intervals (CIs) were provided. Continuous variables were compared between groups using independent *t*-tests, while categorical variables (such as sex distribution) were compared using the chi-square test. A *p*-value of <0.05 was considered statistically significant for all analyses.

## 3. Results

The patient cohort comprised 64 males (35.6%) and 116 females (64.4%). There was no statistically significant difference between Group O and Group A in terms of sex, age (mean age 59.4 ± 14.2 years), body mass index (BMI; mean 26.4 ± 4.9), or prior lumbar surgeries (*p* > 0.05 for all). (Table 1). However, it is important to note that this study is based on a retrospective observational analysis, and patients were not randomly assigned to either group.

In Table 2, the difference in radiation dose per FMBB level between the two groups approached significance, with a *p*-value of 0.06. However, it did not meet the conventional threshold for statistical significance (*p* < 0.05). For the number of FMBB levels blocked, the *p*-value was 0.13, indicating no significant difference between the two techniques.

The results indicate that Group O had a higher total radiation dose and fluoroscopy time compared to Group A. Regarding the dose per one FMBB, Group O had a mean dose of 12.65 ± 7.18 mGy, while Group A had a mean dose of 7.77 ± 4.19 mGy. Regarding fluoroscopy time per one FMBB, Group O had a mean time of 3.33 ± 1.80 s, while Group A had a mean time of 3.19 ± 1.04 s. All differences between the two groups were statistically significant (*p* < 0.001) (Table 3).

The mean total fluoroscopy image count was 91.2 ± 40.3 images for Group O and 53.9 ± 25.6 images for Group A. For 1-level FMBB, the fluoroscopy image count was 10.4 ± 3.7 for Group O and 6.4 ± 2.6 for Group A. The difference between the groups was statistically significant (*p* < 0.001).

The results regarding the performing physicians showed that in Group O, 29 procedures were conducted by professors and 61 by fellows, while in Group A, 25 procedures were performed by professors and 55 by fellows. No statistically significant differences were observed between the groups (*p* = 0.350), indicating that physician distribution across the two techniques was comparable.

For procedures performed by the professors, the mean radiation dose for one FMBB level was 7.95 ± 3.70 mGy (min–max 1.74–16.28). For procedures performed by the fellows, the mean radiation dose for one FMBB level was 11.46 ± 7.80 mGy (min–max 2.26–38.21). The difference in radiation dose per FMBB level between the professors and fellows was statistically significant (*p* < 0.001). Regarding fluoroscopy time for one FMBB level, the professors had a mean time of 3.64 ± 1.59 s, while the fellows had a mean time of 4.71 ± 1.85 s. This difference in fluoroscopy time between the professors and fellows was also statistically significant (*p* < 0.001).

## 4. Discussions

The findings demonstrated significant differences between the techniques and physician performances, with the AP technique and the experienced physician consistently associated with lower radiation exposure and shorter fluoroscopy times.

One of the strengths of our study is that there were no statistically significant differences between the groups in key demographic and clinical variables, including age, sex, and BMI. This is particularly important given that BMI has been shown in the literature to significantly influence both the radiation dose and fluoroscopy time during fluoroscopically guided procedures [11]. Although no significant difference in BMI was observed between the two groups in our study, BMI and other individual factors can still influence radiation exposure. Therefore, we acknowledge that adjusting for BMI could offer additional insight. However, our sensitivity analysis indicated that the presence or absence of adjustments for BMI does not materially affect the results.

Previous lumbar surgeries or additional interventions can complicate the anatomy, potentially leading to increased fluoroscopy times [2]. Furthermore, there were no significant differences between the groups in terms of the number of additional interventions per procedure performed, prior lumbar surgeries, or the specific physicians who performed the procedures. This further strengthens the validity of our findings, as it minimizes potential confounding factors that could otherwise influence the outcomes [12]. The consistency across these variables ensures that our comparison of the oblique and PA techniques is both robust and reliable, providing a clear assessment of their respective impacts on radiation dose.

It is demonstrated that using a direct PA approach significantly reduces scatter radiation and overall radiation dose [4]. Our findings indicated that the AP technique resulted in significantly lower radiation doses compared to the oblique technique. This aligns with previous research suggesting that minimizing the obliquity of the beam and using the PA approach can reduce patient exposure to ionizing radiation during fluoroscopic procedures [13,14]. Lower radiation doses not only benefit patients by reducing their long-term risk of radiation-induced malignancies but also protect clinicians who are repeatedly exposed to scattered radiation during these procedures [15,16].

It is known that simpler, more direct approaches like the PA technique result in shorter fluoroscopy times due to easier target visualization [13]. The AP technique also resulted in significantly shorter fluoroscopy durations compared to the oblique technique, as observed in our findings. The faster completion time with the PA technique could be attributed to its simplicity and direct approach, which facilitates quicker needle placement and reduces the need for repeated imaging adjustments [17]. Shorter fluoroscopy times are advantageous as they not only reduce the procedure time but also enhance reduce radiation exposure.

Additionally, the difference in fluoroscopy times between the professors and fellow physicians was also statistically significant, with the professors completing the procedures in a shorter fluoroscopy time. This finding underscores the importance of experience in reducing procedure duration, which directly correlates with less radiation exposure [18].

Jain et al. (2019) showed that more direct approaches like the PA technique tend to reduce the need for repeated imaging adjustments, resulting in fewer fluoroscopic images [10]. In addition to the reduction in radiation dose, this study also found a significant difference in the total fluoroscopy image count between Group O and Group P. Specifically, the mean total fluoroscopy image count was significantly higher in Group O. This finding underscores the greater efficiency of the AP technique, which requires fewer fluoroscopic images, thereby reducing not only radiation exposure but also potentially decreasing procedure time and improving overall workflow in the clinical setting. These results are consistent with the existing literature, which highlights the benefits of simpler and more direct imaging techniques in minimizing radiation burden and optimizing procedural outcomes [19].

A potential confounding factor that must be considered is the specific skill and experience of each operator in performing one technique over another. While both professors and fellows participated in the procedures, the time required to complete each technique could have been influenced more by the operator’s proficiency with a particular approach rather than their professional rank. Previous studies have highlighted the impact of operator experience on procedure efficiency; for example, a work by Farì et al. demonstrated differences in injection technique performance due to operator expertise. Future studies should account for this variable to better isolate the effects of the fluoroscopic technique itself from operator experience [20].

Experience level is also a known factor influencing radiation exposure and procedure times in fluoroscopy-guided interventions [21]. The variation in radiation dose between physicians further emphasizes the role of experience and technique. Procedures performed by the professors resulted in significantly lower radiation doses than those performed by the fellows. This finding is consistent with studies that highlight how experience and expertise in fluoroscopic techniques contribute to minimizing radiation exposure [22]. The learning curve associated with such procedures suggests that fellows and less experienced clinicians may benefit from targeted training to improve their efficiency and reduce radiation doses [23].

The significant differences observed between the oblique and AP techniques, as well as between physicians with varying levels of experience, have important clinical implications. The AP technique should be considered the preferred method for FMBBs, given its lower radiation dose and shorter procedure time. Moreover, the findings advocate for standardized protocols that emphasize the AP technique, particularly in settings where radiation safety is a priority.

### Limitations

In our study, patients were allocated to the oblique or posterior–anterior technique groups based on clinical considerations and the surgeon’s preference rather than through randomization. This allocation method reflects the real-world clinical decision-making process but also introduces the possibility of selection bias. We acknowledge that the lack of randomization may limit the generalizability of our findings. However, the consistency of the observed differences in radiation doses between the two techniques suggests that the allocation method did not have a substantial influence this study’s primary outcomes. One limitation of this study is the potential for individual or institution-specific practices to influence the outcomes, despite the use of a single radiology technician. Further studies in diverse clinical settings would be beneficial to validate the generalizability of these findings. The retrospective nature of this study might introduce selection bias. Future multicenter studies with a larger sample size and prospective design are needed to validate these findings.

## 5. Conclusions

In conclusion, the AP technique offers significant advantages over the oblique technique in terms of reducing radiation exposure by 39% and fluoroscopy time by 40% during FMBBs. The experience level of the physician also plays a critical role in optimizing these outcomes. These findings should inform clinical practice guidelines and training programs aimed at enhancing the safety and efficiency of fluoroscopy-guided spinal interventions.

## Figures and Tables

**Table 1:** Patient demographics and baseline characteristics.

Characteristic	Group O	Group A	*p*
Number of patients	90	90	-
Age (years), mean ± SD	59.52 ± 13.42	59.20 ± 13.46	0.88
BMI (kg/m^2^), mean ± SD	26.36 ± 3.30	26.53 ± 2.48	0.58
Sex, female–male (*n*) (%)	28 (31.1)–62 (68.9)	36 (40.0)–54 (60.0)	0.28
Prior lumbar surgery	27	22	0.40

Prior lumbar surgery refers to the number of patients in each group who previously underwent surgical interventions on the lumbar spine. This variable is included to assess any potential baseline differences between the groups that might influence the outcomes of the facet medial branch blocks, including radiation exposure and fluoroscopy times. BMI = body mass index; SD = standard deviation.

**Table 2:** Comparison of the number of interventions per procedure and FMBB levels blocked between techniques (mean ± SD).

	Group O	Group A	*p*
Number of interventions per procedure	1.49 ± 0.85	1.39 ± 0.87	0.06
Number of FMBB levels blocked	3.88 ± 0.81	3.68 ± 0.73	0.13

FMBB = facet medial branch block; SD = standard deviation.

**Table 3:** Comparison of radiation dose and procedure time between techniques (mean ± SD).

	Group O	Group A	*p*
Total radiation dose (mGy)	109.15 ± 67.50	65.83 ± 45.49	<0.001
Dose per FMBB level (mGy)	12.65 ± 7.18	7.78 ± 4.19	<0.001
Total fluoroscopy time (seconds)	46.33 ± 19.12	28.09 ± 12.89	<0.001
Time per FMBB level (seconds)	5.34 ± 1.80	3.19 ± 1.04	<0.001
Total fluoroscopy image count	91.2 ± 40.3	53.90 ± 25.60	<0.001

FMBB = facet medial branch block; SD = standard deviation.

## Data Availability

The data supporting the findings of this study have been uploaded to the journal system and are available upon reasonable request from the corresponding author.
